# U3 snoRNA genes are multi-copy and frequently linked to U5 snRNA genes in *Euglena gracilis*^§^

**DOI:** 10.1186/1471-2164-10-528

**Published:** 2009-11-16

**Authors:** J Michael Charette, Michael W Gray

**Affiliations:** 1 Centre for Comparative Genomics and Evolutionary Bioinformatics, Department of Biochemistry and Molecular Biology, Dalhousie University, Halifax, Nova Scotia, Canada; 2 Current address: Department of Molecular Biophysics & Biochemistry, Yale University School of Medicine, Sterling Hall of Medicine, 333 Cedar St, Room SHM C-114, PO Box 208024, New Haven, CT 06520-8024, USA; 3 Correspondence address: Room 8-F2, Sir Charles Tupper Medical Building, Dalhousie University, 5850 College Street, Halifax, B3H 1X5, Nova Scotia, Canada

## Abstract

**Background:**

U3 snoRNA is a box C/D small nucleolar RNA (snoRNA) involved in the processing events that liberate 18S rRNA from the ribosomal RNA precursor (pre-rRNA). Although U3 snoRNA is present in all eukaryotic organisms, most investigations of it have focused on fungi (particularly yeasts), animals and plants. Relatively little is known about U3 snoRNA and its gene(s) in the phylogenetically broad assemblage of protists (mostly unicellular eukaryotes). In the euglenozoon *Euglena gracilis*, a distant relative of the kinetoplastid protozoa, Southern analysis had previously revealed at least 13 bands hybridizing with U3 snoRNA, suggesting the existence of multiple copies of U3 snoRNA genes.

**Results:**

Through screening of a λ genomic library and PCR amplification, we recovered 14 U3 snoRNA gene variants, defined by sequence heterogeneities that are mostly located in the U3 3'-stem-loop domain. We identified three different genomic arrangements of *Euglena *U3 snoRNA genes: *i*) stand-alone, *ii*) linked to tRNA^Arg ^genes, and *iii*) linked to a U5 snRNA gene. In arrangement *ii*), the U3 snoRNA gene is positioned upstream of two identical tRNA^Arg ^genes that are convergently transcribed relative to the U3 gene. This scenario is reminiscent of a U3 snoRNA-tRNA gene linkage previously described in trypanosomatids. We document here twelve different U3 snoRNA-U5 snRNA gene arrangements in *Euglena*; in each case, the U3 gene is linked to a downstream and convergently oriented U5 gene, with the intergenic region differing in length and sequence among the variants.

**Conclusion:**

The multiple U3 snoRNA-U5 snRNA gene linkages, which cluster into distinct families based on sequence similarities within the intergenic spacer, presumably arose by genome, chromosome, and/or locus duplications. We discuss possible reasons for the existence of the unusually large number of U3 snoRNA genes in the *Euglena *genome. Variability in the signal intensities of the multiple Southern hybridization bands raises the possibility that *Euglena *contains a naturally aneuploid chromosome complement.

## Background

*Euglena gracilis *is a free-living protist, distantly related to kinetoplastid protozoons [1], and whose RNA molecular biology displays a number of peculiar features. One example is the cytoplasmic ribosome of *Euglena *and the genes encoding its constituent rRNAs. Typically in eukaryotes, hundreds to thousands of copies of the rRNA operon are tandemly encoded on linear chromosomes. In *Euglena*, a single copy of the rRNA operon is carried on an 11-kbp extrachromosomal circular plasmid that is present in high copy number (estimates range between 800 and 4000 per cell) and that replicates autonomously, with few if any integrated chromosomal rRNA genes [2-5]. Furthermore, a number of additional, novel internal transcribed spacer sequences in the *Euglena *28S rDNA are excised during pre-rRNA processing [6]. Consequently, the mature LSU rRNA, which typically consists of two stable species (5.8S plus 28S rRNA), is composed of 14 rRNA pieces (5.8S plus 13 28S 'fragments') in *Euglena *[7]. A similar, but less extensive pre-rRNA processing in trypanosomes yields a seven-fragment LSU rRNA [8-10]. Despite the extreme fragmentation of the *Euglena *LSU rRNA, the individual pieces are able to associate in *trans *[11] to form functional ribosomes [12-15]. Furthermore, ongoing mapping of *O*^2'^-methylribose and pseudouridine positions (M.N. Schnare & M.W. Gray, unpublished) suggests that the constituent LSU rRNA is the most highly modified rRNA of any organism examined to date [16].

The small nucleolar RNAs (snoRNAs) constitute a very large family of small RNAs present in the nucleolus as ribonucleoprotein complexes. Most snoRNAs guide the site-specific formation of *O*^2'^-methylribose or pseudouridine modifications in rRNAs and snRNAs [17-19]. Some of the processing and cleavage events required for the liberation of mature rRNAs from the pre-rRNA transcript are mediated by a subset of snoRNAs. One such processing snoRNA, the box C/D snoRNA U3, was the first snoRNA identified [20] and has since become the most extensively studied.

U3 snoRNA consists of 5'- and 3'-domains separated by a hinge region [21]. The 5'-domain contains sequence elements that are complementary to regions of the 5'-external transcribed spacer (ETS) of the pre-rRNA, as well as to the 5'-end of the 18S rRNA [22-26]. Base-pairing interactions between the pre-rRNA and its complementary regions in U3 snoRNA guide, by a complex and incompletely understood mechanism that includes the participation of U14 [27,28] and U17/snR30 [29-31] snoRNAs, the multiple sequence-specific pre-rRNA cleavage events that eventually liberate the mature 5'-end of the 18S rRNA [22-26,32]. Additional base-pairing interactions occur between the central hinge region of U3 snoRNA and the 5'-ETS of the pre-rRNA [5,33-35]. These multiple base-pairing interactions may also confer on U3 snoRNA a chaperone-like activity in the co-transcriptional folding of the 18S rRNA [25]. The 3'-domain of U3 snoRNA, important in protein binding and RNA stability, contains box C/D sequence elements characteristic of this snoRNA family [21,24].

U3 snoRNA associates with at least 43 proteins (the four box C/D snoRNA proteins plus 25 U-three-associated proteins (Utps) and 14 others) to form a large ribonucleoprotein complex termed the SSU processome [21,36]. In 'Miller chromatin spreads' of actively transcribed rRNA genes visualized by electron microscopy, this massive 80S complex of ~2.2 MDa corresponds to the terminal knobs observed on the 5'-ends of growing pre-rRNA 'Christmas trees' [36,37].

Information about the many players, such as U3 snoRNA and its protein components, and the multiple, intricate and highly coordinated events in rRNA processing and ribosome biogenesis, has accumulated largely from studies in the phylogenetically narrow grouping of animals and yeasts. Therefore, our current understanding of ribosome biogenesis may not be truly representative of the many potentially different strategies used by phylogenetically disparate organisms. For this reason, the idiosyncratic features of RNA biology in *E. gracilis *coupled with its key phylogenetic placement make this organism a potentially informative alternative model system for the study of rRNA maturation, ribosome biogenesis and the many RNA and protein components that participate in this highly complex process.

*Euglena *U3 snoRNA [38] is a 180-nt species exhibiting the sequence motifs typical of U3 snoRNAs [21]. While considerably smaller than its homologues in yeast and vertebrates (333 nt, *S. cerevisiae *[21]; 217 nt, human [39]), *Euglena *U3 snoRNA is comparable in size to its trypanosomatid relatives (143 nt, *T. brucei *[40]). Unexpectedly, whereas U3 snoRNA is encoded by a single-copy gene in all examined trypanosome species [40-42], heterogeneities observed in RNA sequencing along with multiple hybridizing bands in Southern analysis [38] strongly suggested that U3 snoRNA is a multi-copy gene in the *Euglena *genome. In trypanosomatids, the genomic neighborhood of the U3 snoRNA locus is particularly rich in genes for other small RNAs, such as tRNAs and snRNAs [41-43]. Furthermore, in these organisms, the expression of the U3 snoRNA gene is dependent upon the presence of a closely linked, upstream and divergently oriented tRNA gene [44,45]. Thus, we reasoned that an exploration of the genomic contexts of the *Euglena *U3 snoRNA gene loci would uncover both commonalities and differences with respect to the trypanosomatid scenario. In addition, insights might be gained both into the evolution of U3 snoRNA and the functional architecture of the *Euglena *genome.

Here, we describe the results of a comprehensive analysis of *E. gracilis *U3 snoRNA genes and their genomic contexts. We confirm that unlike the trypanosomatid case, where U3 snoRNA is a single-copy gene, the *Euglena *U3 snoRNA is encoded by a multigene family comprising at least 14 members. As in trypanosomes, most *Euglena *U3 snoRNA genes are located near genes for other small RNAs, such as tRNAs and U5 snRNA. However, notable differences between the *Euglena *and the trypanosomatid arrangements are evident, the evolutionary and functional implications of which are considered here.

## Results

### Multiple Southern hybridizing bands imply that the gene encoding U3 snoRNA is multi-copy in the *Euglena *genome

**Figure 1:**
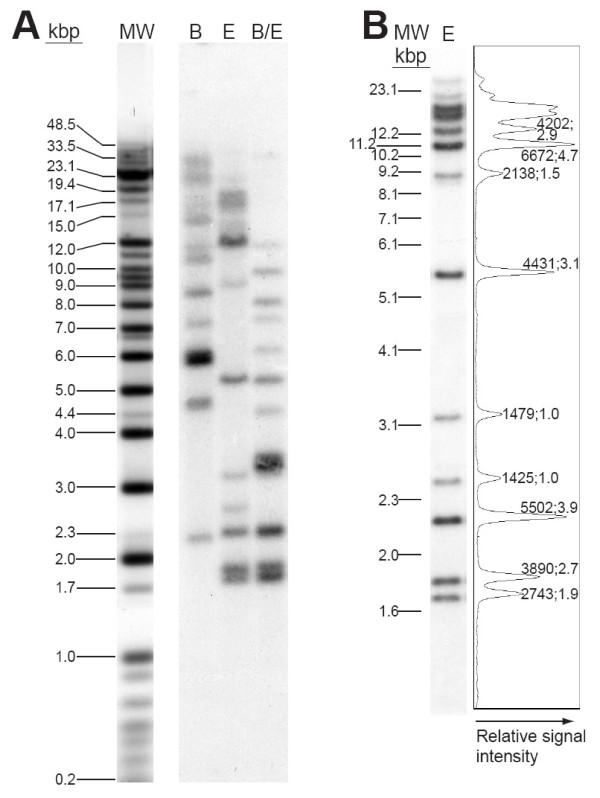
**Southern analysis of restriction endonuclease-digested *Euglena *DNA demonstrating multiple bands hybridizing with a U3 snoRNA gene probe**. **(A) ***Euglena *DNA was digested with *Bam*H1 (B), *Eco*R1 (E) or with both (B/E) endonucleases, two restriction enzymes that do not have recognition sequences within the *Euglena *U3 snoRNA gene. Restriction fragments were resolved by electrophoresis prior to Southern transfer and hybridization with a *Euglena *U3 snoRNA probe, all as described in Methods. The exact number of hybridizing fragments in the 10 to 38.5 kbp size range is difficult to determine owing to the limited resolution of this size range on standard agarose gels. (MW; a mixture of Invitrogen™ λ DNA/*Hin*dIII, 1 Kb Plus and λ DNA/High Molecular Weight Markers) **(B) **Densitometric analysis confirms that the multiple hybridizing bands display unequal hybridization signal intensities. *Euglena *DNA, digested with *Eco*R1 (E), was resolved by electrophoresis in a 0.7% agarose/1× TAE gel prior to Southern transfer and hybridization. The results of densitometric analysis using ImageJ [97] are shown to the right of the sample lane, with peak areas corresponding to the relative signal intensities of the labeled bands. For the indicated bands, the first number gives the area under the peak while the second number represents signal intensity normalized to that of the 2.5-kbp band (signal intensity 1425, the lowest in the lane).

A complex and reproducible pattern of multiple hybridizing bands is produced in Southern hybridization experiments with a *Euglena *U3 snoRNA gene probe (Fig. 1A), implying that this gene is present in multiple copies in the *Euglena *genome. The resulting hybridization patterns show at least 13 hybridizing *Eco*R1, *Bam*H1 or *Eco*R1/*Bam*H1 fragments, ranging in size from ~1.8 to ~38.5 kbp.

Unexpectedly, the multiple U3-hybridizing fragments showed reproducible differences in signal intensity (Fig. 1B), with this apparent non-stoichiometry being confirmed by densitometric analysis. Comparison within each sample lane revealed a number of bands with very similar signal intensities, including the 2.5-, 3.1-, and 9.2-kbp bands (signal intensities 1425, 1479, and 2138, respectively; Fig. 1B). Bands at 1.8, 5.5, and 12.2 kbp also displayed very similar signal intensities (3890, 4431, and 4202, respectively). On the other hand, differences in relative signal intensity within a sample lane are clearly illustrated by comparison of the 1.7-, 1.8- and 2.2-kbp bands, whose signal intensities were 1.9×, 2.7×, and 3.9× that of the 2.5-kbp band (2743, 3890 and 5502, respectively, vs.1425). Similar results have been obtained with other hybridization probes (see below). The reproducibility of these results suggests that they are not attributable to incomplete restriction endonuclease digestion of the DNA or to uneven transfer of restriction fragments from gel to membrane prior to hybridization.

### Three different U3 snoRNA gene arrangements identified in a λ library of *Euglena *genomic DNA

**Figure 2:**
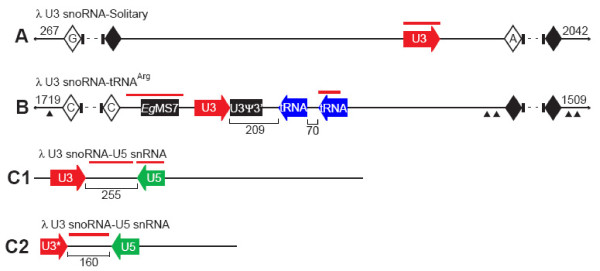
**Three different U3 snoRNA gene arrangements in λ clones of *Euglena *genomic DNA**. Clone maps of (**A**) λ U3-Solitary (total size ~12 kbp; 4824 bp sequenced; U3-containing portion 2515 bp), (**B**) λ U3-tRNA^Arg ^(total size ~14 kbp; 6096 bp sequenced; U3-containing portion 2868 bp) and (**C1 and C2**) λ U3-U5 (total sizes 1772 and 516 bp, respectively) are shown. Terminal sequences of specified length (bp) do not contain any features of interest. Sizes of the intergenic spacers are indicated by the numbers between the various genes. Red lines demarcate the positions of probes used in Southern hybridization experiments. Repetitive elements, such as the *Euglena Eg*MS7 microsatellite [47] (filled rectangle), the U3 snoRNA pseudo-3'-end repeat (U3Ψ3' filled rectangle) and repetitive sequences also found within certain *Euglena *γ-tubulin introns (filled triangles) are indicated. Solid diamonds denote simple sequence repeats that precluded effective primer design for additional primer walking. Open diamonds with letters denote homopolymer runs (A, C or G) that prematurely terminated sequencing. Due to the technical challenges posed by the many repetitive sequence elements, the λ U3-Solitary and λ U3-tRNA^Arg ^clones were not sequenced in their entirety; dashed lines denote unsequenced portions. In the λ U3-U5-C2 clone, U3* refers to a 5' truncation of the U3 gene as a result of cloning. Clone maps not drawn to scale.

To investigate further the genomic organization of *Euglena *U3 snoRNA, we screened a λ library of *Euglena *genomic DNA using a *Euglena *U3 snoRNA gene probe, retrieving several U3-hybridizing λ clones. Sequence walking in conjunction with BLASTn analysis revealed four unique U3 snoRNA gene variants in three different genomic contexts (Fig. 2A-C): *i*) a stand-alone gene, *ii*) linkage to tRNA genes, and *iii*) linkage to a U5 snRNA gene.

A solitary U3 snoRNA gene arrangement was identified in a large 12-kbp genomic fragment (Fig. 2A). No additional, recognizable genes or sequence elements were found by BLASTn analysis.

A U3-hybridizing λ clone was found to encode a U3 snoRNA gene neighbored downstream by two identical arginine tRNA genes, both encoded in the opposite transcriptional orientation relative to the U3 snoRNA gene (Fig. 2B). The coding regions of the U3 snoRNA gene and first tRNA^Arg ^gene are separated by 209 bp, whereas the two tRNA^Arg ^genes are 70 bp apart. No additional genes were found by BLASTn analysis. This clone contains many short simple-sequence elements, also identified in other λ clones, such as homopolymer and dinucleotide repeats. In addition, unusual repetitive sequence elements are present, such as repeat sequences that are also present within the introns of the *Euglena *γ-tubulin gene paralogs [46]. The region upstream of the U3 snoRNA gene also contains a *Euglena *microsatellite sequence, *Eg*MS7 [47]. Finally, a 52-nt region, consisting of the 3'-end and putative transcription termination sequence of this particular U3 snoRNA gene variant, has evidently been duplicated. The resulting sequence consists of a full-length U3 snoRNA gene and transcription termination signal followed by the 3'-terminal 22-nt of the U3 snoRNA gene (nt 159 to 180) and a nearly identical copy of the transcription termination signal.

**Figure 3:**
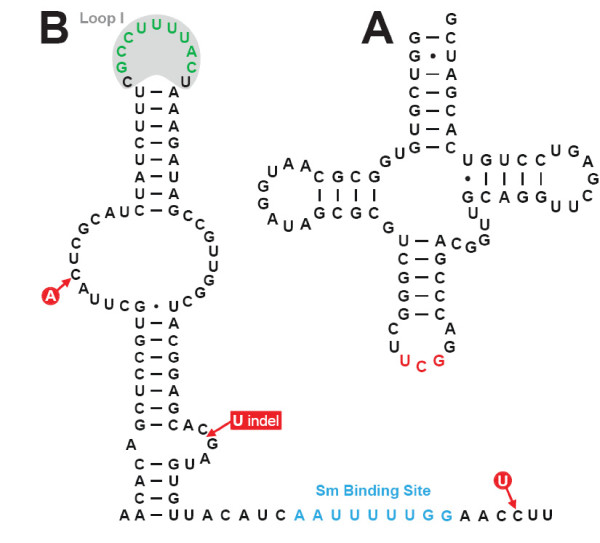
**(A) The *Euglena *tRNA^Arg ^adopts a conventional clover-leaf secondary structure**. The anticodon nucleotides, UCG, are highlighted in red. Watson-Crick base-pairing interactions are depicted as dashes (-) and G/U pairings with dots (•). **(B) **The *Euglena *U5 snRNA displays conventional secondary structure features. The phylogenetically conserved and functionally essential exon-interacting nucleotides (green) of loop I (gray background) and the Sm binding site (blue) are highlighted. Nucleotide heterogeneities and an indel in the gene variants are indicated in red.

The identified tRNA^Arg ^gene is unremarkable in sequence and secondary structure (Fig. 3A). It adopts a conventional clover-leaf secondary structure, displays the expected D and TΨC loops, and possesses a small loop in the variable region.

Two different U3-hybridizing λ clones were identified in which the U3 snoRNA gene is neighbored downstream by a convergently oriented U5 snRNA gene (Fig. 2C1/C2). No additional genes were detected. While the U3 snoRNA and U5 snRNA sequences in the two clones are highly similar, the size (255 and 160 bp) and sequence of the U3 snoRNA-U5 snRNA intergenic spacers are very different (see below). Furthermore, the sequence of the regions downstream of the U5 snRNA genes is equally dissimilar. As with the other λ clones, the two U3-U5 genomic inserts contain many short repetitive sequence elements and stretches of extreme nucleotide bias (e.g., hompolymeric stretches of up to 12 C or G residues).

As with most protist U snRNAs, the *Euglena *U5 snRNA gene sequence was previously unknown. Its nucleotide sequence and secondary structure (Fig. 3B) display features present in U5 snRNAs from other organisms. The *Euglena *U5 snRNA is 98 nt in length, the position of its 5'-end inferred by comparison with other U5 snRNA sequences. The precise 3'-end was determined by 3' RACE analysis and by chemical sequencing of the RNA (data not shown). The secondary structure consists, in its 5'-region, of a stem-loop region punctuated by a central bulge. The 11-nt terminal loop I contains the invariant 9-nt sequence (5'-GCCUUUUAC-3') known to interact with exon sequences at the 5'- and 3'-splice sites [48]. The 3'-region contains a conventional Sm binding site. Notably, a small stem-loop structure, typically present near the 3'-end of U5 snRNAs, is not found in the *Euglena *U5 snRNA.

### Southern analysis suggests that *Euglena *U3 snoRNA genes are frequently linked to U5 snRNA genes

Although intensive screening of the *Euglena *λ genomic library identified only four different U3 snoRNA genes in three distinct genomic contexts, Southern analysis of *Euglena *genomic DNA revealed at least 13 U3-hybridizing bands. Because we could not account for many U3 snoRNA genes (and their genomic arrangements), Southern analysis was performed to determine whether additional variants of the linkages identified in the λ genomic fragments are present in the *Euglena *genome.

**Figure 4:**
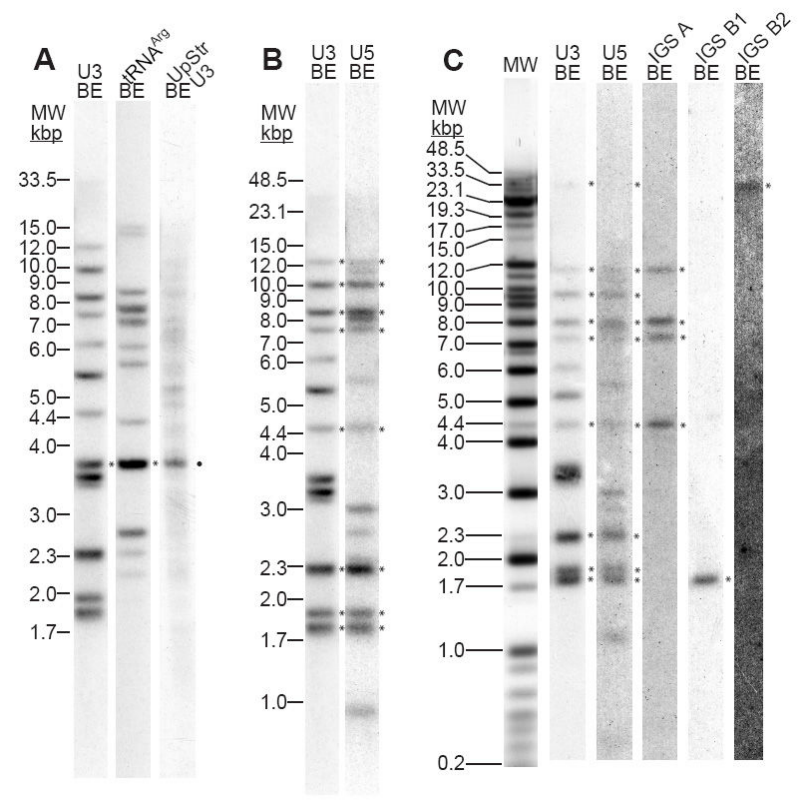
**Southern analysis of *Euglena *DNA hydrolyzed with *Bam*H1 + *Eco*RI (BE) reveals few U3-tRNA^Arg ^but multiple U3-U5 gene linkages**. **(A) **Hybridization with probes corresponding to the *Euglena *U3 and tRNA^Arg ^genes, and to a region upstream of the U3 gene (UpStr U3) but containing the *Euglena *microsatellite *Eg*MS7 [47] sequence, all as described in Methods. MW: a mixture of Invitrogen™ λ DNA/*Hin*dIII, 1 Kb Plus and λ DNA/High Molecular Weight Markers. **(B) **Hybridization with a *Euglena *U5 probe. **(C) **Probes corresponding to the IGS sequence of the U3-U5 linkage families A and B (both sub-families B1 and B2) confirm the genomic PCR results. Bands co-hybridizing with U3, U5 and IGS probes are indicated by the asterisks. The IGS probes frequently hybridized to large restriction fragments and gave low signal intensities, despite long autoradiographic exposures. Accordingly, in the case of the IGS lanes only, the contrast was uniformly adjusted over the entire lane to aid in the identification of hybridizing band(s).

Southern analysis of *Euglena *genomic DNA using a tRNA^Arg ^gene probe identified multiple hybridizing bands (12 in *Bam*HI/*Eco*RI, ranging in size from 2.1 kbp to 16 kbp; Fig. 4A), suggesting that the tRNA^Arg ^gene is also multi-copy in the *Euglena *genome. This result was not unexpected, considering that tRNA genes frequently constitute large, multigene families.

A single band, co-hybridizing with the U3 and tRNA^Arg ^probes (indicated by the asterisks in Fig. 4), is suggestive of a single U3-tRNA^Arg ^gene linkage in the *Euglena *genome. Other members of the tRNA^Arg ^gene family do not appear to be similarly linked to U3 snoRNA genes.

The authenticity of the apparent U3-tRNA^Arg ^co-hybridization was further substantiated by the observation that a probe derived from the region upstream of the U3 gene in the U3-tRNA^Arg ^λ clone (Fig. 2B) predominantly labeled the band that hybridized with both the U3 and tRNA^Arg ^probes (•, Fig. 4A). This probe also contains the *Euglena *microsatellite sequence [47] mentioned above, which likely explains the relatively high level of background hybridization seen in this particular case.

Southern analysis of *Euglena *genomic DNA with a U5 gene probe identified ~14 hybridizing fragments, ranging in size from 0.9 kbp to 13 kbp. (Fig. 4B). Thus, U5 snRNA is also encoded by multiple genes in the *Euglena *genome.

Comparison of the U5 Southern hybridization result with the U3 one revealed at least eight co-migrating hybridization bands (asterisks, Fig. 4B). Thus, the majority of U5 snRNA genes, though not all, appeared to be linked to U3 snoRNA genes in the *Euglena *genome.

In addition, as observed with the U3-hybridizing bands, the U5-hybridizing bands also showed reproducible differences in hybridization intensity. Furthermore, the relative signal intensities within the U5 pattern co-vary with those within the U3 pattern.

### Genomic PCR confirms multiple U3 snoRNA-U5 snRNA gene linkages in the *Euglena *genome

**Figure 5:**
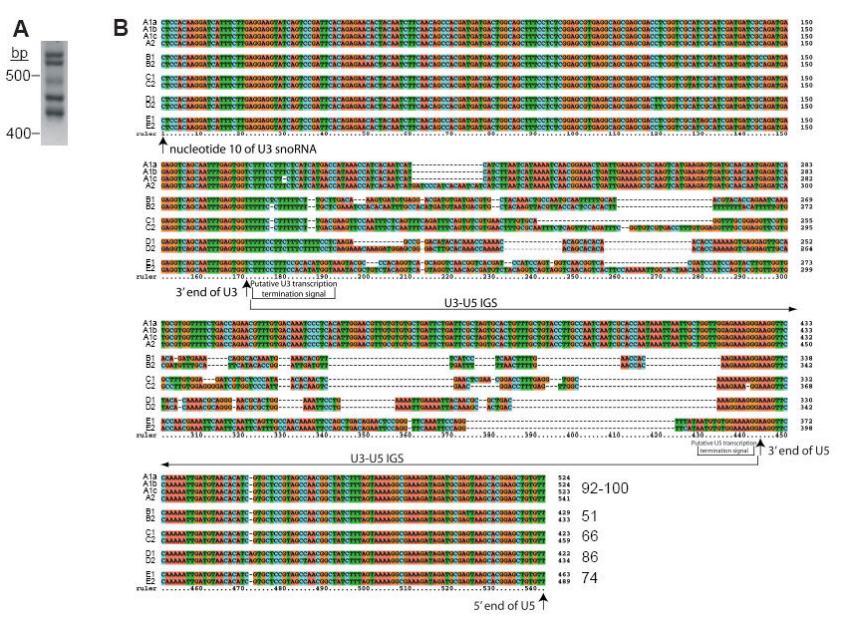
**Multiple amplification products from *Euglena *genomic PCR reveal U3 snoRNA-U5 snRNA gene linkages that cluster into related families and sub-families**. **(A) **PCR amplification of total *Euglena *DNA with primers designed to the 5'-regions of the convergently oriented U3 snoRNA and U5 snRNA genes (o*Eg*U3-F1 and o*Eg*U5-F1; see Additional file 2) yields multiple amplification products ranging in size from ~425 to ~550 bp. **(B) **Alignment of PCR products demonstrates high sequence conservation of the U3 snoRNA and U5 snRNA genes, both within a linkage family and between linkage families. Numbers at the ends of the alignments indicate the percent sequence identity (% ID) among family members within the IGS, excluding the U3 and U5 coding sequences and putative transcription termination signals. Family members that are identical within the IGS may nevertheless display nucleotide heterogeneities within the flanking U3 or U5 genes. The alignment begins at nt 10 of the U3 snoRNA gene, owing to the location of the PCR primer.

To examine putative U3-U5 gene linkages in detail, we used a genomic PCR strategy to amplify, clone and sequence U3-U5 intergenic spacers (IGS) as well as the flanking U3 and U5 genes. As expected from the sequence of the two U3-U5 λ clones and from the results of Southern analysis, multiple PCR products were obtained (Fig. 5A). To ensure complete coverage of all U3-U5 linkages, a total of 122 clones, from five different PCR-generated libraries, were sequenced and analyzed, with multiple clones of each linkage being identified. Thus, nucleotide heterogeneities between the different U3-U5 linkages are considered legitimate and not attributable to *Taq*-induced errors or to PCR-mediated recombination [49-51].

The cloned U3-U5 PCR products ranged in size from 422 to 541 bp. Detailed sequence analysis identified a total of 12 unique U3-U5 linkages. Comparison of the unique sequences revealed that the multiple U3 snoRNA sequences are highly similar, as are the multiple U5 snRNA sequences, the members of each group displaying only limited sequence heterogeneities (Fig. 5B). However, substantial variability is seen in the size and sequence of the IGS separating the U3 and U5 genes. Despite this variability, regions of sequence similarity within the IGS suggest that the gene linkages may be related (e.g., compare the IGS in A1a and A1b, Fig. 5B). Thus, the 12 unique U3 snoRNA-U5 snRNA gene linkages appear to form five families that can be further divided into sub-families (Fig. 5B).

U3-U5 family A, representing the linkage having the longest IGS sequence, comprises four members. Based on sequence similarity, the members of this family can be divided into two sub-families, A1 and A2, with the A1 subfamily being further divided into A1a, A1b and A1c. Members of the A family show the highest level of identity, exhibiting only minor nucleotide changes and a 17-nt indel in the IGS sequence. The A1a PCR linkage (Fig. 5B) corresponds to the U3-U5 linkage identified in the λ genomic clone C1 (Fig. 2).

The B-linkage family contains two members, B1 and B2. The IGS sequences of the two linkages show the lowest level of sequence identity of any of the linkage families. The relatedness of the members of the B-linkage family is based on the presence of short regions of sequence identity, which are punctuated by regions of nucleotide divergence. Short regions of apparent sequence similarity at both ends of the IGSs, immediately downstream of the 3'-ends of the U3 and U5 genes, may correspond to transcription termination signals, in which case these regions do not actually contribute to linkage family relatedness. The B1 PCR linkage (Fig. 5B) corresponds to the U3-U5 linkage identified in the λ genomic clone C2 (Fig. 2).

The C-, D-, and E-linkage families each contain two members, C1 and C2, D1 and D2, and E1 and E2. Each linkage family exhibits regions of sequence similarity interrupted by regions of nucleotide difference and by indels. Overall, the level of sequence identity in the members of the C, D and E linkage families is intermediate between that of the highly similar A family and the very divergent B family. Some of the U3-U5 linkages were confirmed by Southern hybridization analysis (Fig. 4C).

A similar genomic PCR approach was used to search for other arrangements of linked U3-U5 genes, such as divergently (head-to-head) and similarly (tail-to-head) oriented genes. The possible presence of tandem U3 snoRNA genes was also investigated. No linkages of these types were found.

### A revised secondary structure model for *Euglena *U3 snoRNA

**Figure 6:**
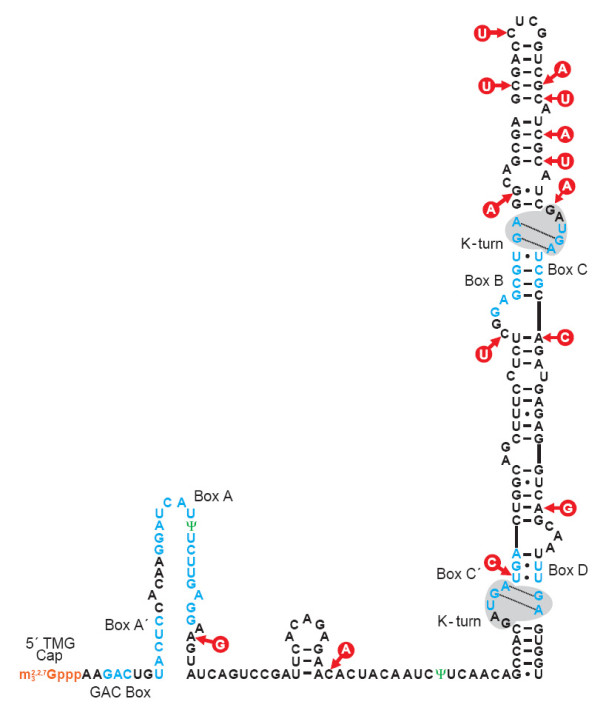
**Revised secondary structure model of the *Euglena *U3 snoRNA**. In this proposed model, the 5'-trimethylguanosine cap (5'-TMG cap) is indicated in orange, the phylogenetically conserved and functionally essential box GAC, A', A, C', B, C, and D elements are shown in blue. Pseudouridine (Ψ) residues are indicated in green. Nucleotide heterogeneities identified in the *Euglena *U3 snoRNA gene variants are denoted by the red circles. Nucleotides forming a K-turn motif are highlighted on a gray background. Conventional Watson-Crick base-pairing interactions are depicted as dashes (-) and G/U pairings are identified by dots (•). An alignment of known U3 snoRNA sequences from representative organisms is shown in Additional file 1.

The nucleotide heterogeneities identified in the U3 variants prompted a re-assessment of the likely secondary structure of *Euglena *U3 snoRNA. A recent phylogenetic comparison of U3 sequences from representative taxa [21] has uncovered additional conserved sequence elements and RNA motifs that are also present in the *Euglena *U3 snoRNA (Fig. 6). An alignment of known U3 snoRNA sequences from representative organisms is shown in Additional file 1. Furthermore, information from mutational studies [21,33] and from *in vivo *chemical and enzymatic structure-probing experiments [24,52] has resulted in significant revision of the proposed general secondary structure of U3 snoRNA. Accordingly, a revised conserved sequence element and secondary structure model for *Euglena *U3 snoRNA is presented here (Fig. 6).

Sequence elements in the 5' region of U3 are known to interact with the 5' external transcribed spacer (5'-ETS) of the pre-rRNA and with the 5' region of the 18S rRNA. The first such element in U3 snoRNA is the GAC box, followed by the A' box and then the A box [21]. In the identified *Euglena *U3 genes, a single nucleotide heterogeneity has been mapped to this region. The heterogeneity, an A-to-G transition, is located downstream of the A box sequence.

The central hinge region of the *Euglena *U3 snoRNA contains a small stem-loop structure consisting of a 3-bp stem with a 5-nt loop. As in other organisms [22], potential base-pairing interactions may occur between the *Euglena *U3 hinge domain (both the 5' and 3' regions) and the 5' external transcribed spacer (5' ETS) of the pre-rRNA [5]. In the multiple *Euglena *U3 genes, a sequence heterogeneity is located in the 3' hinge region. This A-to-C transversion is located immediately downstream of the small stem-loop structure.

The 3'-terminal domain of the *Euglena *U3 snoRNA contains conserved sequence elements, boxes C', B, C and D, known to interact with RNA-binding proteins [53,54]. This domain consists of an extended stem-loop structure punctuated by a number of bulge-loop elements. In our revised model, box C' and box D elements are juxtaposed in the secondary structure context, as are box B and box C elements. The box elements are mainly single-stranded, corresponding to small bulge-loop structures; however, some regions may form short base-pairing interactions. More importantly, alternative base-pairing interactions in the 3'-terminal domain allow for the formation of two kink-turn (K-turn) RNA motifs [54-56] within the juxtaposed box C'/D and B/C elements.

Nearly all of the sequence heterogeneities identified in the multiple *Euglena *U3 snoRNA variants (12 of the 14) are located in the 3'-extended stem-loop domain. Ten of the 12 sequence heterogeneities are transitions, vs. two transversions. The majority of nucleotide heterogeneities in helical regions result in shifts from Watson-Crick base-pairings (U-A and G-C) to non-canonical interactions (three U•G, one G•U). Three heterogeneities occur in single-stranded regions, none of which is part of a single-stranded conserved box element. Two nucleotide heterogeneities, present in the same U3 snoRNA variant near the terminal stem-loop structure, form compensatory base changes (C-G to U-A) and thus maintain base-pairing interactions. Two additional nucleotide heterogeneities map to the terminal base pairs of short stem regions, immediately adjacent to the opening or closing of bulge-loop structures. Since neither of these heterogeneities maintains the terminal base-pairing interactions of the stems, a slight expansion of the adjacent bulge-loop structures results. One of the two heterogeneities is located within the box C' element, adjacent to the 5+2 motif of the K-turn [55,56]. Only one heterogeneity, located in a short stem region, disrupts a standard base-pairing interaction. In sum, the distribution of the 12 sequence heterogeneities identified in the 3'-extended stem-loop domain of *Euglena *U3 snoRNA results in minimal changes to the overall secondary structure of the region.

## Discussion

### In Euglenozoa, U3 snoRNA genes are frequently linked to genes for other small RNAs

**Figure 7:**
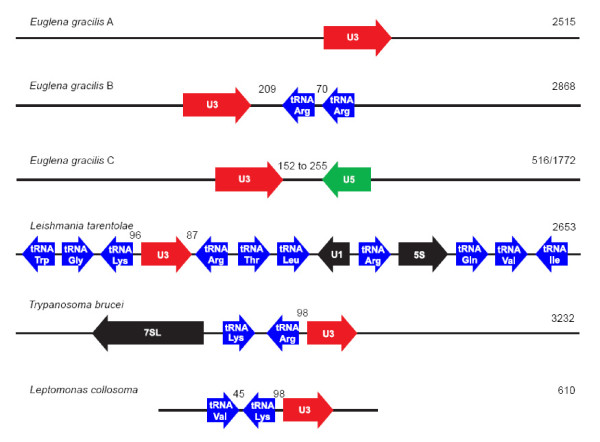
**Organization of U3 snoRNA genes within Euglenozoa**. The U3 snoRNA genes are shown in red, U5 snRNA gene in green, tRNA genes in blue and all other genes are shown in black. *Euglena gracilis *A, B and C correspond to the arrangements depicted in Fig. 2. Numbers shown between the various genes correspond to the sizes of the intergenic spacers separating the two genes. Numbers on the right hand of the figure indicate the size (bp) of the respective genomic region. Note that this figure is not drawn to scale. Organisms represented include *Euglena gracilis *(this study), *Leishmania tarentolae *[GenBank:L20948], *Trypanosoma brucei *[GenBank:X57047] and *Leptomonas collosoma *[GenBank:L32919].

In one of three patterns of U3 snoRNA gene organization identified here, a U3 snoRNA gene is linked to two identical, downstream and convergently oriented (relative to the U3 snoRNA gene) tRNA^Arg ^genes in the *Euglena *genome (Fig. 2B). Close linkage of U3 snoRNA and tRNA genes has been found in a number of trypanosomatids, members of the kinetoplastid protozoa, which are the closest relatives of the euglenids. The most dramatic example is in *Leishmania tarentolae*, where a 2.7-kbp genomic region encodes the U3 snoRNA gene along with genes for 10 tRNAs, U1 snRNA and 5S RNA (Fig. 7) [41]. Sequence for a portion of the corresponding region in *L. major *(GenBank AQ843909) reveals the same gene content organized in the same manner. In both *Leishmania *cases, the U3 snoRNA gene is neighbored by a single, downstream, convergently oriented tRNA^Arg ^gene (possessing the same anticodon, UCG, as in the *Euglena *case).

In *Trypanosoma brucei*, the U3 snoRNA gene is neighbored upstream by a divergently oriented tRNA^Arg ^gene (having a different anticodon, ACG); no proximal genes are encoded downstream of the *T. brucei *U3 snoRNA gene (Fig. 7) [42,57]. In *Leptomonas collosoma*, a divergently oriented tRNA^Lys ^gene is also encoded upstream of the U3 snoRNA gene [43] (no sequence is available downstream). Similarly, an upstream, divergently oriented tRNA^Lys ^gene neighbors the U3 snoRNA gene in both *L. tarentolae *and *L. major*, in addition to the downstream, convergently oriented tRNA^Arg ^gene noted above. In all of these trypanosomatid cases, the IGS separating the U3 and tRNA genes is 71 to 106 bp in size, compared to 209 bp in the *Euglena*. In the phylogenetically unrelated ciliate *Tetrahymena thermophila*, a U3 snoRNA gene is also neighbored by an upstream, identically oriented tRNA^Lys ^gene. However, in this case, the U3-tRNA IGS is nearly 500 bp in size [58]. Evidently, close physical linkage of U3 snoRNA and tRNA genes is a widespread phenomenon within the trypanosomatids, with similar linkages in *Euglena *and in *Tetrahymena*.

The most striking observation to emerge from the present study is the multiplicity of distinct U3 snoRNA genes and gene arrangements in the *Euglena *genome, in stark contrast to what is seen in trypanosomatids [40-43]. In addition to the U3-tRNA^Arg ^linkage, we document here 12 distinct examples of U3 snoRNA-U5 snRNA gene linkage in the *Euglena *genome. In all these examples, the U5 snRNA gene is located downstream and in the opposite transcriptional orientation relative to the U3 snoRNA gene (Fig. 2 and 5). To date, no linked U3-U5 genes have been identified in other organisms. Furthermore, with the exception of tRNA genes, U3 snoRNA genes have not been found directly linked to other genes encoding small RNAs.

Genes specifying modification-guide box C/D and box H/ACA snoRNAs are frequently clustered, including in trypanosomatids [59,60] and in *Euglena *[61]. So far, however, U3 snoRNA genes have not been found linked to modification-guide snoRNA genes in any eukaryote, and this also appears to be the case in *Euglena*.

### Influence of U3 snoRNA gene organization on U3 snoRNA gene expression

The expression of U3 snoRNA genes in trypanosomatids and other eukaryotes has been extensively studied. In all instances examined, trypanosomatid [62], other protist [58] and plant [63,64] U3 snoRNA genes are transcribed by RNA polymerase III (RNAP III). Based on the sister-group relationship between kinetoplastids and euglenids [1], U3 snoRNA is similarly assumed to be synthesized by RNAP III in *Euglena*, although this inference remains to be confirmed experimentally. In all cases, U3 snoRNA genes are transcribed from their own promoter and U3 transcripts do not appear to be part of larger, polycistronic transcripts.

In trypanosomatids, the expression of U3 snoRNA is dependent on the linked, upstream, divergently oriented tRNA gene. Two extragenic regulatory elements (A and B boxes) required for trypanosome U3 snoRNA gene expression [44] correspond to the tRNA D and TΨC loops, which are known to serve generally as intragenic RNAP III promoter elements for tRNA genes. Furthermore, the spacing of the A and B boxes relative to each other and to the U3 snoRNA gene is critical. As noted above, the distance between the upstream tRNA gene and the U3 snoRNA gene varies within a narrow range (93-105 bp) in trypanosomatids (Fig. 7). Transcription factor TFIIIC is known to bind to the B box element of the tRNA gene, whereupon it recruits transcription factor TFIIIB, which in turn sequesters RNAP III for transcription initiation [65,66] In yeast, transcription of tRNA genes by RNAP III prevents nucleosome assembly in the immediate vicinity of these genes, and concomitant nucleosome-mediated repression [67]. Thus, the binding of TFIIIC to the B box is postulated to play an indirect role, through chromatin remodeling, in the expression of trypanosomatid tRNA-linked U3 snoRNA genes [44,62]. Whether the downstream tRNA^Arg ^genes play a similar role in U3 snoRNA transcription in *Euglena *remains to be determined. The *Euglena *U3 snoRNA-tRNA genes are not as closely linked (209 bp IGS) as they are in trypanosomatids (93-105 bp IGS). However, the presence of two tRNA^Arg ^genes in the *Euglena *case might result in more efficient recruitment of TFIIIC to this site and a parallel chromatin remodeling effect over a larger region. The fact remains that most U3 snoRNA genes in *Euglena *are evidently not linked to tRNA genes, so any parallel with U3 snoRNA gene expression in trypanosomatids must necessarily be limited.

### Gene duplication in the generation of multiple U3 snoRNA-U5 snRNA gene linkages in *Euglena*

The 12 different U3 snoRNA-U5 snRNA gene linkages described here cluster into five discrete families, based on sequence conservation within the IGS (Fig. 5B). In the 'A' family, three linkage variants form a sub-family (A1a-A1c) while a fourth linkage is the sole member of the second sub-family (A2). Linkage families B, C, D, and E contain two U3 snoRNA-U5 snRNA gene linkages each. No regions of sequence conservation common to all 12 linkage variants are evident within the IGS.

At this point, the origin and generation of the multiple U3 snoRNA-U5 snRNA gene linkages remains a matter of conjecture. It is not unreasonable to suppose that the current arrangement arose via duplication of one or a few ancestral U3 snoRNA-U5 snRNA gene linkages. The pathway of formation and fixation of the ancestral U3-U5 gene linkage(s) is unclear, but the linkage *per se *presumably was generated by random gene shuffling and perhaps maintained by a favorable effect on gene expression. Multiple rounds of locus, chromosome and/or genome duplication followed by sequence divergence likely then created the observed linkage families and sub-families.

In support of this proposal, accumulating evidence from sequence analysis indicates that parts of the *Euglena *genome are highly repeated and that the genome may be evolutionarily plastic. An arrangement similar to the U3-U5 linkage case has been described for the *Euglena *spliced leader RNA (SL RNA) gene, which is linked to a 5S rRNA gene [68]. As with the U5 snRNA gene, some SL RNA genes are dispersed throughout the genome; however, the majority are linked to an identically oriented 5S rRNA gene. The linkage is encoded within a tandemly repeated 0.6-kbp unit, and it is estimated that up to 300 SL RNA-5S rRNA gene linkages are present in the *Euglena *genome. In the absence of large-scale linkage data, we do not know whether the U3 snoRNA-U5 snRNA gene linkages described here might be similarly present in the *Euglena *genome as repeated arrays, although PCR amplification results would seem to rule out any closely linked U3-U5 units.

Limited information on gene copy number for other *Euglena *snRNAs gives a contrasting picture. Southern hybridization analysis of *Euglena *DNA with a *Euglena *U1 snRNA [69] gene probe yields a single hybridizing band, a result substantiated by PCR amplification and the sequencing of several independent *Euglena *U1 snRNA clones, which revealed no nucleotide heterogeneities (unpublished results). In contrast, when 3' RACE-based PCR amplification was used to obtain the sequence of the *Euglena *U4 snRNA [70], at least four sequence variants were obtained (unpublished results). Additional evidence for the repetitive nature of portions of the *Euglena *genome has come from studies of *Euglena *modification-guide (box C/D and box H/ACA) snoRNAs, where many cases of multi-copy, linked snoRNA genes have been found [16,61]. Similarly, on-going bioinformatic screens of *Euglena *cDNAs have revealed multiple allelic variants of typically single-copy ribosomal protein genes (unpublished results). Thus, it appears that many, though not all, genes are multi-copy in the genome of *Euglena*.

### Are U3 snoRNA genes spatially organized in the nucleus?

A recent model for the spatial organization of RNAP III-transcribed genes in the nucleus may be relevant to the organization and expression of U3 snoRNA genes in *Euglena*, trypanosomatids and some other organisms. This model stems from *in situ *hybridization studies suggesting that the 274 tRNA genes of yeast, although dispersed throughout the linear map of the genome, are localized to the nucleolus [71,72]. This situation is analogous to the three-dimensional co-localization of the multiple rDNA genes in the nucleolus. Accordingly, it has been proposed that chromosomal loci encoding tRNA genes also associate in three-dimensional space within the nucleus. Such an arrangement could conceivably lead to the formation of a tRNA transcription and processing center enriched in RNAP III, transcription, and processing factors [71,73]. Experimental evidence suggests that U3 snoRNA genes in human cells may associate in three-dimensional space with coiled bodies in the nucleus [39,74]. Thus, the three-dimensional clustering of genes transcribed by RNAP III could promote the formation of 'transcription territories' [75] that function to more efficiently recruit transcription complexes to the region, thereby maintaining a high level of gene expression. Although it is not known whether this scenario applies to *Euglena *U3 snoRNA genes, the suggestion is a plausible one considering that *Euglena *U3 snoRNA genes are likely transcribed by RNAP III and for the most part are linked either to tRNA genes or to the RNAP III-transcribed U5 snRNA gene.

### Why so many U3 snoRNA genes in the *Euglena *genome?

The *Euglena *genome encodes at least 14 different U3 snoRNA genes. While U3 snoRNA is an essential gene, it is unclear why the *Euglena *genome would encode so many copies, considering that the U3 snoRNA gene is single-copy in trypanosomatid protozoa [40-43]. At least part of the explanation for this evident expansion of U3 snoRNA genes in *Euglena *may have to do with the unusual features of its rRNA genes and mature rRNA species.

In a *Euglena *cell, a single unit of linked rRNA genes is encoded on each of ~1000-4000 copies of an extrachromosomal plasmid-like DNA species [2-5], in contrast to the arrangement of chromosomally integrated tandem rDNA arrays in most other eukaryotes. Electron micrographs show an unusual, extensively segmented nucleolar structure in *Euglena*, which might reflect the organization of the individual plasmid-like rDNA elements into a number of distinct, higher order clusters within the nucleus. (In *Entamoeba *sp., the rDNA is similarly encoded on an extrachromosomal DNA element that is able to induce formation of a dispersed nucleolar structure located at the nuclear periphery [76].) If U3 snoRNA genes are also physically and functionally localized at or near the nucleolus, the additional numbers of U3 snoRNA genes seen in *Euglena *may be required to accommodate an atypical nucleolar organization of its rDNA genes, thereby more efficiently coordinating U3 snoRNA expression and function with rRNA synthesis and processing.

Another consideration is that, relative to the situation in a typical eukaryote, many additional spacer sequences are removed during pre-rRNA processing in *Euglena*, resulting in a naturally and highly fragmented 28S rRNA equivalent [6,7]. One suggestion is that *Euglena *U3 snoRNA might participate in these additional pre-rRNA processing events [38]. Alternatively, the additional processing steps might result in the production of a relatively higher proportion of defective ribosomes than in other eukaryotes, in which case a relatively larger number of pre-rRNA transcripts would presumably need to be processed in order to maintain an adequate number of functional ribosomes in the cell. This requirement would in turn necessitate a greater number of U3 snoRNA molecules, a requirement that presumably could be achieved by encoding and expressing an expanded number of U3 snoRNA genes.

A problem with the above suggestions is that trypanosomatid LSU rRNAs are also fragmented, although not as extensively as those in *Euglena *[8-10]. Nevertheless, as noted above, the U3 snoRNA gene is single copy in trypanosomatid genomes [40-43]. Thus, it appears unlikely that the multi-copy nature of *Euglena *U3 snoRNA genes could be a direct consequence of the particularities of rRNA processing in *Euglena*.

### U3 snoRNA genes and the *Euglena *genome

Considering the long history of *Euglena gracilis *as a 'laboratory workhorse', surprisingly little is known about its genome. The varying intensities of U3 snoRNA-hybridizing bands in Southern blots of *Euglena *DNA suggests that the actual number of U3 snoRNA genes may be substantially higher than the 14 we have documented here. Technical considerations in restriction endonuclease digestion, transfer of restriction fragments and subsequent hybridization have been eliminated as possible sources of artifact contributing to hybridization variability, based on the reproducibility of the results under different experimental conditions.

Aneuploidy, in which a cell contains different numbers of one or more chromosomes, appears to be the most likely explanation for the varying intensities of hybridizing bands. In many organisms, aneuploidy is associated with genome instability, as in human Trisomy-21 (Down's syndrome) and many cancers. However, aneuploidy has been postulated to occur normally in a number of protists [77] and in fungi [77-79], although in these cases it does not appear to result in genome instability.

Aneuploidy has been reported in a number of trypanosomatids, including *Trypanosoma cruzi *[77,80-83] and *Leishmania *[84]. In such cases, results similar to those presented here have been obtained. In pulsed field gel electrophoretic analysis of *T. cruzi *DNA, the ethidium bromide staining intensities of different chromosomal bands varies within single samples. Furthermore, DNA-content variability of up to 70% has been observed in cells derived from a single clone. There is as yet no direct evidence for aneuploidy in *Euglena*; however, the demonstrated occurrence of aneuploidy in trypanosomatids, in conjunction with the results presented here, makes aneuploidy a distinct possibility in *Euglena*.

## Conclusion

The comprehensive analysis reported here has revealed multiple U3 snoRNA genes in the protist *Euglena gracilis*, in three distinct genomic arrangements: *i*) stand-alone, *ii*) linked to two tandem, identical tRNA^Arg ^genes, and *iii*) linked to a U5 snRNA gene. The multiple U3 snoRNA-U5 snRNA gene linkages, which cluster into distinct families based on sequence similarities within the intergenic spacer, presumably arose by genome, chromosome, and/or locus duplications. We suggest that the evident expansion of U3 snoRNA genes in *Euglena*, compared to its kinetoplastid distant relatives, may have to do with the unusual features of *Euglena *rRNA genes and mature rRNA species and/or a highly recombinogenic genome. In view of the variability in the signal intensities of the multiple bands consistently observed in our Southern hybridization experiments, we further raise the possibility that *Euglena *contains a naturally aneuploid chromosome complement.

## Methods

### Extraction of total DNA from *Euglena *gracilis

A streptomycin-bleached, aplastidic variant of *Euglena gracilis *[2], derived from the UCLA variety of *E. gracilis *strain Z, was grown in 1-liter cultures of medium [85] at room temperature with gentle agitation. The medium was modified by addition of ethanol to 30 mM as a carbon source [2], CoCl_2 _(1.3 mg/l) and Na_2_MoO_4 _(0.2 mg/l) in place of Co(NO_3_)_2 _and H_2_MoO_4_, respectively, and adjusted to pH 6.5 with phosphoric acid [86]. Cultures were harvested at mid- to late-log phase (after 4-5 days of growth), at an OD_600 _between 0.8 and 1.0.

Total *Euglena *DNA was prepared using a modified detergent/chloroform/phenol extraction procedure. *Euglena *cultures, held on ice for 30 min, were centrifuged at 3,520 *g *for 20 min at 4°C. The cells were subsequently washed in a total of 150 ml cold Extraction Wash Buffer (EWB = 25 mM EDTA-Tris (pH 8.5): an EDTA solution titrated to pH 8.5 with solid Tris base), pooled, recovered by centrifugation at 3,520 *g *for 10 min and resuspended to a final volume of 10 ml with room temperature EWB.

Cells were lysed by the addition of 2.5 ml 25% SDS. The solution was gently mixed until homogeneous, after which the lysate volume was adjusted to 25 ml with room temperature EWB. To the lysate, 4 ml 8 M sodium perchlorate was added and the solution was gently mixed to homogeneity. Nucleic acids were extracted twice with chloroform:isoamyl alcohol (24:1) and precipitated with an equal volume of room temperature 2-propanol. High-molecular-weight DNA was preferentially recovered by spooling onto a glass rod, then washed with 80% ethanol, briefly dried and dissolved in 10 ml TE (10 mM Tris-HCl (pH 7.6), 0.1 mM EDTA). Once re-dissolved, the DNA was further deproteinized by phenol-cresol extraction until no material was visible at the interface. The DNA was precipitated from the aqueous phase with ethanol, washed, briefly dried and dissolved in 1.0 ml TE.

Contaminating RNA in the DNA preparation was removed by RNase treatment. DNA was preferentially recovered by polyethylene glycol (PEG) precipitation [87], which involved adjusting the solution to final concentrations of 0.5 M NaCl and 10% PEG and incubating on ice for 30 min. The PEG-precipitated DNA was recovered by centrifugation at 11,180 *g *for 15 min at 4°C. The DNA was washed twice with 80% ethanol, briefly dried and redissolved in TE. Residual PEG was removed by additional phenol extractions and ethanol precipitations.

### DNA amplification by polymerase chain reaction

PCR amplifications (50 μl reactions) consisted of 200 μM of each dNTP, 1× ThermoPol buffer (New England BioLabs), 2.5 U *Thermus aquaticus *DNA polymerase, 20 pmol each oligonucleotide primer [see Additional file 2] and 100-500 ng total *Euglena *DNA. In certain cases, amplifications were improved by the use of sheared genomic DNA (50 passes through a 29-gauge syringe). Thermal cycling conditions consisted of 96°C for 5 min followed by 35 cycles of *i*) 95°C for 40 sec, *ii*) 55°C for 40 sec and *iii*) 72°C for 50 sec with a final extension at 72°C for 7 min. PCR parameters for products intended for cloning in the TOPO TA Cloning^® ^system (Invitrogen™) consisted of 30 amplification cycles and a final extension at 72°C for 30 min, as recommended by the manufacturer.

### Purification of DNA fragments by gel electrophoresis

Small DNA fragments (<1 kbp) were resolved by electrophoresis in 1-2.5% (w/v) low-melting-point (LMP) agarose gels containing 1× TAE (100 mM Tris, 0.1 mM Na_2_•EDTA, titrated to pH 8.0 with glacial acetic acid). Gel slices (0.4-0.55 g) were transferred to 2-ml microcentrifuge tubes. The slices were crushed then melted by incubation at 65°C for 30 min. The melted gel was incubated with 0.5 vol. phenol-cresol, pre-warmed at 65°C, and incubated for 5 min at 65°C with frequent vigorous agitation. The supernatant was recovered by centrifugation and extracted twice more with phenol. After the addition of 0.1 vol. 3 M NaOAc, the supernatant was extracted four additional times with phenol at room temperature, or until no material was visible at the interface. DNA was precipitated with ethanol (facilitated by the addition of 5 μl 0.25% linear polyacrylamide carrier [88]), recovered by centrifugation, washed in 75% ethanol, dried and redissolved in 5-10 μl TE.

### Cloning of PCR products, plasmid minipreps and sequence analysis

When necessary, PCR products were purified by gel electrophoresis using the LMP agarose/hot phenol method, or with the Sephaglas™ BandPrep kit (Amersham Biosciences), and cloned using the TOPO TA Cloning^® ^kit (Invitrogen™). Plasmid DNA was prepared using the QIAprep^® ^Spin Miniprep kit (Qiagen) and sequenced in-house. Large insert clones were sequenced by primer walking [see Additional file 2]. Sequence chromatograms were analyzed and contig assembly was performed using the Staden Package software suite [89]. Gene sequences were identified by BLAST [90] sequence similarity searches at GenBank http://www.ncbi.nlm.nih.gov/blast/. Sequence alignments were generated using ClustalX [91] and manually edited with BioEdit [92]. RNA secondary structures were drawn with XRNA [93].

### Southern hybridization analysis

Total *Euglena *DNA was digested with the specified restriction endonuclease (10 U/μg DNA, in the presence of DTT and BSA) for 5 hr at 37°C, to ensure complete digestion. For these experiments, we used a combination of *Bam*H1 and *Eco*R1, two restriction endonucleases that do not have recognition sequences within the *Euglena *U3, U5 or tRNA^Arg ^genes or in the U3-U5 IGS sequences examined. Restriction fragments, 10-13 μg/lane, were resolved by electrophoresis in a 0.5% agarose/1× TAE gel at 1 V/cm for 20 hr. DNA fragments were depurinated and denatured [94,95] prior to capillary transfer in 0.4 M NaOH to a charged nylon membrane (GeneScreen Plus^®^, NEN^® ^Life Science Products) according to the manufacturer's alkaline transfer protocol. Following overnight transfer, the membrane was neutralized in 0.2 M Tris-HCl (pH 7.6)/2× SSC (1× SSC = 150 mM NaCl, 15 mM sodium citrate) for 5 min, washed in 2× SSC for 5 min and baked at 80°C for ~8 hr. The nylon membrane was cut into strips corresponding to groups of duplicate sample lanes.

Cloned Southern hybridization probes were either excised from their plasmid vector by restriction digestion or amplified by PCR from the plasmid insert, with LMP agarose/hot phenol-based gel purification when necessary. Approximately 100 ng of each probe was labeled by DNA synthesis in the presence of random hexamer primers [96] and [α-^32^P]dATP.

Membranes were soaked in 6× SSC for 5 min, then washed for >30 min in 6× SSC at hybridization temperature (42°C). The blots were pre-hybridized for 4-18 hr in Southern hybridization solution (5× Denhardt's solution, 5× SSC, 25 mM Na_2_HPO_4_, 25 mM NaH_2_PO_4_, 180 μg/ml sheared and denatured herring testes DNA, 50% formamide, 1% SDS) [38,95], then hybridized for 18-22 hr. The membranes were washed at 42°C by five 10-min incubations in 2× SSC/0.1% SDS and once or twice for 15 min in 0.1× SSC/0.1% SDS. The relative signal intensities of the multiple hybridizing bands were quantified by densitometric analysis using ImageJ [97] to determine the area under each peak.

### Screening of an *E. gracilis *λ Genomic DNA Library

An *E. gracilis *λ genomic DNA library, constructed in the λBlueSTAR™ vector (Novagen), was screened by plaque lift (as per the membrane manufacturer's protocol; Magna Lift, Osmonics) and hybridization with a *Euglena *U3 snoRNA gene probe. Hybridization and washing conditions were identical to those described for Southern hybridization. Positive λ plaques were excised *in vivo *into plasmid clones by Cre-mediated recombination and transferred to *E. coli *DH5α cells, as described in the library kit protocol. Plasmid DNAs were prepared and sequenced as described above.

### Accession Numbers

The *Euglena *sequences presented here have been deposited under the following accession numbers: λ U3 snoRNA-solitary [GenBank:GQ338155, GenBank:GU080027 and GenBank:GU080026]; λ U3 snoRNA-tRNA^Arg ^[GenBank:GU080028, GenBank:GU080030 and GenBank:GU080029]; λ U3 snoRNA-U5 snRNA C1 [GenBank:GU080031]; λ U3-U5 C2 [GenBank:GU080032]; U3 snoRNA-U5 snRNA linkage family A1a, contained within λ genomic clone C1 [GenBank:GU080031]; U3-U5 linkage A1b [GenBank:GU080033]; U3-U5 linkage A1c [GenBank:GU080034]; U3-U5 linkage A2 [GenBank:GU080035]; U3-U5 linkage B1, contained within λ genomic clone C2 [GenBank:GU080032]; U3-U5 linkage B2 [GenBank:GU080036]; U3-U5 linkage C1 [GenBank:GU080037]; U3-U5 linkage C2 [GenBank:GU080038]; U3-U5 linkage D1 [GenBank:GU080039]; U3-U5 linkage D2 [GenBank:GU080040]; U3-U5 linkage E1 [GenBank:GU080041]; U3-U5 linkage E2 [GenBank:GU080042].

## Authors' contributions

JMC performed the experimental work, interpreted the results and drafted the manuscript. MWG conceived the project, provided advice and interpretation, and contributed to and revised the manuscript. Both authors read and approved the final manuscript.

## Note

^§ ^This paper is dedicated to the memories of Claude Charette, the father of JMC, and Grazyna Tokarczyk, a technician in the lab, both of whom passed away while this work was in progress.

## Supplementary Material

Additional file 1 **U3 snoRNA sequence alignment**. An alignment of known U3 snoRNA sequences from representative organisms. Conserved sequence features in U3 snoRNA, boxes GAC, A', A, C', B, C, and D are shown, along with regions of the alignment corresponding to the central hinge and 3'-hairpin domains. Representative organisms include: *Arabidopsis thaliana *[GenBank:X52629, nt 325-541], *Chlamydomonas reinhardtii *[GenBank:AJ001179, nt 171-392], *Crithidia fasciculata *[GenBank:AF277396], *Dictyostelium discoideum *[GenBank:V00190, nt 62-271], *Euglena gracilis *[GenBank:U27297], *Homo sapiens *[GenBank:M14061, nt 277-493], *Leishmania major *[GenBank:AQ843909], *Leishmania tarentolae *[GenBank:L20948, complement of nt 2128-1984], *Leptomonas collosoma *[GenBank:L32919, nt 391-533], *Mus musculus *[GenBank:X63743, nt 815-1027], *Saccharomyces cerevisiae *[GenBank:X05498], *Schizosaccharomyces pombe *[GenBank:X56982, nt 37-291], *Tetrahymena thermophila *[GenBank:X71349], *Triticum aestivum *[GenBank:X63065, nt 858-1063], *Trypanosoma brucei *[GenBank:M25776], *Trypanosoma cruzi *[GenBank:AAHK01001296, nt 4547-4689] and *Xenopus laevis *[GenBank:X07318, nt 1-219]. Click here for file

Additional file 2 **DNA oligonucleotides**. A list of DNA oligonucleotide primers used for PCR amplifications, DNA sequencing and 3' RACE. Click here for file
