# Supplementary figures and images for: U3 snoRNA genes are multi-copy and frequently linked to U5 snRNA genes in Euglena gracilis§
Source: BMC Genomics. 2009 Nov 16;10:528. doi: 10.1186/1471-2164-10-528 (PMC2784804; doi:10.1186/1471-2164-10-528)

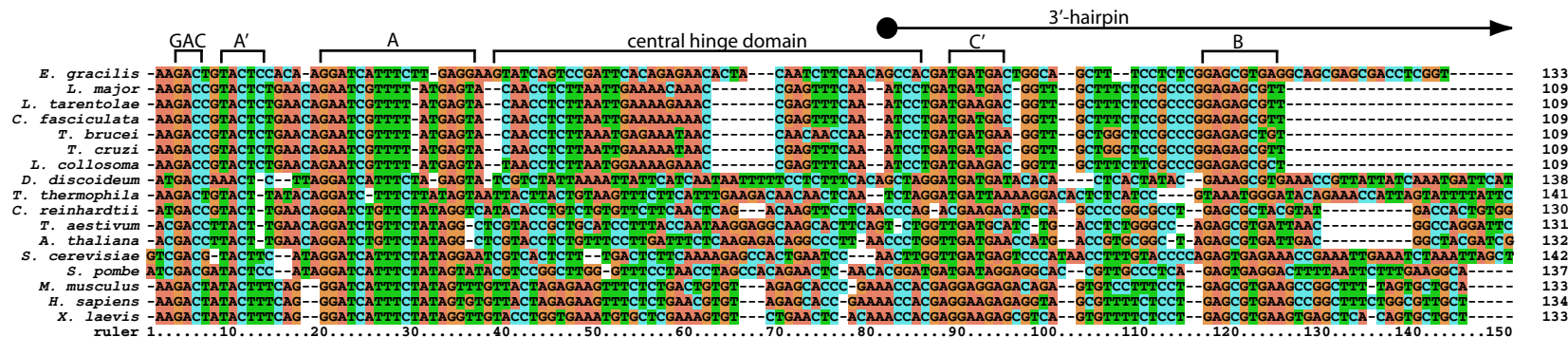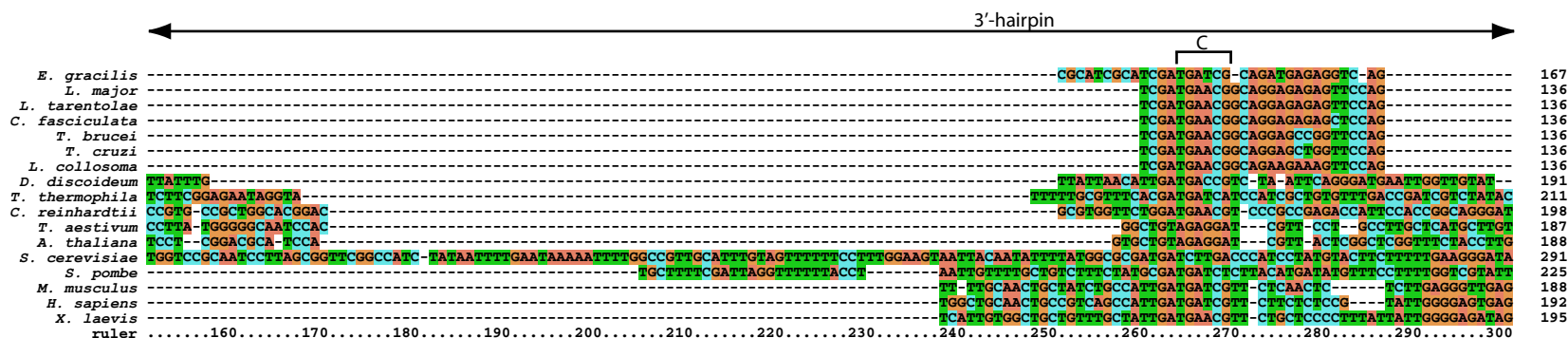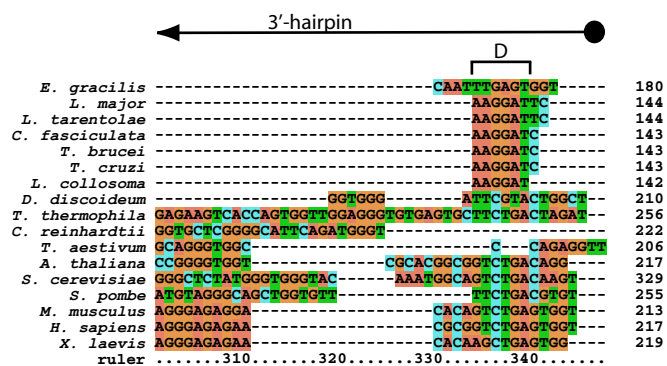

Supplement: Additional file 1 — U3 snoRNA sequence alignment. An alignment of known U3 snoRNA sequences from representative organisms. Conserved sequence features in U3 snoRNA, boxes GAC, A', A, C', B, C, and D are shown, along with regions of the alignment corresponding to the central hinge and 3'-hairpin domains. Representative organisms include: Arabidopsis thaliana [GenBank:X52629, nt 325-541], Chlamydomonas reinhardtii [GenBank:AJ001179, nt 171-392], Crithidia fasciculata [GenBank:AF277396], Dictyostelium discoideum [GenBank:V00190, nt 62-271], Euglena gracilis [GenBank:U27297], Homo sapiens [GenBank:M14061, nt 277-493], Leishmania major [GenBank:AQ843909], Leishmania tarentolae [GenBank:L20948, complement of nt 2128-1984], Leptomonas collosoma [GenBank:L32919, nt 391-533], Mus musculus [GenBank:X63743, nt 815-1027], Saccharomyces cerevisiae [GenBank:X05498], Schizosaccharomyces pombe [GenBank:X56982, nt 37-291], Tetrahymena thermophila [GenBank:X71349], Triticum aestivum [GenBank:X63065, nt 858-1063], Trypanosoma brucei [GenBank:M25776], Trypanosoma cruzi [GenBank:AAHK01001296, nt 4547-4689] and Xenopus laevis [GenBank:X07318, nt 1-219]. [file 1471-2164-10-528-S1.PDF]
