# Supplementary material for: U3 snoRNA genes are multi-copy and frequently linked to U5 snRNA genes in Euglena gracilis§
Source: BMC Genomics. 2009 Nov 16;10:528. doi: 10.1186/1471-2164-10-528 (PMC2784804; doi:10.1186/1471-2164-10-528)
Supplement: Additional file 2 — DNA oligonucleotides. A list of DNA oligonucleotide primers used for PCR amplifications, DNA sequencing and 3' RACE. [file 1471-2164-10-528-S2.DOC]

| **Oligonucleotide** | **Comments** | **Sequence (5′ to 3′)** |
| --- | --- | --- |
| o*Eg*U3-F1 | Near 5′ end of U3 snoRNA; used for PCR, sequencing and 3′ RACE.^38^ | CTCCACAAGGATCATTTCTTGAGG |
| o*Eg*U3-F2 | Near 3′ end of U3 snoRNA; used for sequencing and 3′ RACE. | GATGAGAGGTCAGCAATTTGAGTGG |
| o*Eg*U3-F3 | Near 3′ end of U3 snoRNA; used for sequencing and 3′ RACE. | GATGAGAGGTCAGCAATTTGAGTGGTCTTTCC |
| o*Eg*U3-R1 | Near 3′ end of U3 snoRNA; used for PCR and sequencing.^38^ | CCACTCAAATTGCTGACCTCTCATC |
| o*Eg*U3-R2 | Near 5′ end of U3 snoRNA; used for sequencing and northern hybridizations.^38^ | CTCTGTGAATCGGACTGATACTTC |
| o*Eg*U3-R3 | Near 3′ end of U3 snoRNA; used for PCR. | CCACTCAAATTGCTGACCTCTC |
| o*Eg*U5-F1 | 5′ end of U5 snRNA; used for PCR, sequencing and 3′ RACE. | GCAACACAGCTCCGTGCTTACTCG |
| o*Eg*U5-F2 | Middle of U5 snRNA; used for 3′ RACE. | CTAAAGATAGCCGTTGGCTACGGAGC |
| o*Eg*U5-R1 | 3′ end of U5 snRNA; used for PCR, sequencing and northern hybridizations. | GTTCCAAAAATTGATGTAACACATCG |
| o*Eg*U5-R2 | 3′ end of U5 snRNA; used for PCR. | GTTCCAAAAATTGATGTAACACATC |
| o*Eg*U5-R3 | 5′ end of U5 snRNA; used for PCR and sequencing. | GATAGATGCGAGTAAGCACGGAGC |
| o*Eg*tRNA-Arg-F1 | 5′ end of tRNA^Arg^; used for PCR and sequencing. | GTCGTGTGGCGCAATGGATAG |
| o*Eg*tRNA-Arg-F2 | Middle of tRNA^Arg^; used for PCR and sequencing. | GCAATGGATAGCGCGTCGGGCTTCG |
| o*Eg*tRNA-Arg-R1 | 3′ end of tRNA^Arg^; used for PCR, sequencing and northern hybridizations. | GATCGTGACAGGACTCGAAC |
| o*Eg*tRNA-Arg-R2 | Middle of tRNA^Arg^; used for PCR and sequencing. | CGTGACAGGACTCGAACCTGCAACCG |
| o*Eg*U3U5IGS-A1-F1 | 5′ end of U3-U5 IGS linkage A1; used for PCR and sequencing. | GACCATAAACCATCACAATCATC |
| o*Eg*U3U5IGS-A1-R1 | 3′ end of U3-U5 IGS linkage A1; used for PCR and sequencing. | GTGCGATTGATTGGCAAGGTACAGC |
| o*Eg*U3U5IGS-B1-F1 | 5′ end of U3-U5 IGS linkage B1; used for PCR and sequencing. | GCTTGCCAAAGTGATGTGAGG |
| o*Eg*U3U5IGS-B1-R1 | 3′ end of U3-U5 IGS linkage B1; used for PCR and sequencing. | CTTGTGGTTCAAAAGTTGAGG |
| o*Eg*U3U5IGS-B2-F1 | 5′ end of U3-U5 IGS linkage B2; used for PCR and sequencing. | GCTCGAAATCCACACAATTTGCC |
| o*Eg*U3U5IGS-B2-R1 | 3′ end of U3-U5 IGS linkage B2; used for PCR and sequencing. | GGAACTTTCCTTTTCTTGTGG |
| o*Eg*U3U5IGS-C1/2-F1 | 5′ end of U3-U5 IGS linkage C1 and C2; used for PCR and sequencing. | CTTGACGAAGTTCCAATTTCTC |
| o*Eg*U3U5IGS-C1/2-R1 | 3′ end of U3-U5 IGS linkage C1 and C2; used for PCR and sequencing. | CCNTTTYTTTGCCAMCTCAAAGGTCCG |
| o*Eg*U3U5IGS-D2-F1 | 5′ end of U3-U5 IGS linkage D2; used for PCR and sequencing. | CCTCAAGAAACAAAGATGGAGCGGG |
| o*Eg*U3U5IGS-D2-R1 | 3′ end of U3-U5 IGS linkage D2; used for PCR and sequencing. | CCCTTCCTTTGTCAGTGCTTTG |
| o*Eg*U3U5IGS-E1-F1 | 5′ end of U3-U5 IGS linkage E1; used for PCR and sequencing. | CACATGGTAAGTACGCCCACAGG |
| o*Eg*U3U5IGS-E2-F1 | 5′ end of U3-U5 IGS linkage E2; used for PCR and sequencing. | CCACATATGGTAAATACGCTGTC |
| o*Eg*U3U5IGS-E1/2-R1 | 3′ end of U3-U5 IGS linkage E1 and E2; used for PCR and sequencing. | CCTTTTCCACACATTATRAACCTGG |
| o*Eg*λU3solitary-3FW1 | Sequence walking primer for lambda clones. | GTTGGTTCCAATAACCTGCC |
| o*Eg*λU3solitary-3FW2 | Sequence walking primer for lambda clones. | GGACTCGTGGGTATTCAAAGG |
| o*Eg*λU3solitary-3FW3 | Sequence walking primer for lambda clones. | GATGCGCGATGGTCGCATTGAGC |
| o*Eg*λU3solitary-3RW1 | Sequence walking primer for lambda clones. | TACTGGGAAGAGGTGGACACTC |
| o*Eg*λU3solitary-3RW2 | Sequence walking primer for lambda clones. | CTCGCATTGATCTTCGCGTCTGGC |
| o*Eg*λU3solitary-3RW3 | Sequence walking primer for lambda clones. | CTCCTTGATCCAACTTCATGCTGCC |
| o*Eg*λU3-tRNA-Arg-U3FW1 | Sequence walking primer for lambda clones. | GCTGAATCATGCAACACAGAGC |
| o*Eg*λU3-tRNA-Arg-U3FW2a | Sequence walking primer for lambda clones. | ATGCGCAGATCTCGCGTTCG |
| o*Eg*λU3-tRNA-Arg-U3FW2b | Sequence walking primer for lambda clones. | GGACTAGTTGAAGAGAGTGAGCG |
| o*Eg*λU3-tRNA-Arg-U3FW2c | Sequence walking primer for lambda clones. | CCAGAATAAATCCATAAATTGCC |
| o*Eg*λU3-tRNA-Arg-U3FW3 | Sequence walking primer for lambda clones. | GCTGTTTCTGGGCCGAAAATGAGCG |
| o*Eg*λU3-tRNA-Arg-U3RW1 | Sequence walking primer for lambda clones. | ATATCTCACCCACCATGCACTTCG |
| o*Eg*λU3-tRNA-Arg-U3RW2 | Sequence walking primer for lambda clones. | GGCAATTTATGGATTTATTCTGG |
| o*Eg*λU3-tRNA-Arg-U3RW4 | Sequence walking primer for lambda clones. | GAACAACGATGCTTTACCAAACC |
| o*Eg*λU3U5A-T7RW1 | Sequence walking primer for lambda clones. | ATCCTCGCCTTGCACACACTGC |
| o*Eg*λU3U5A-U3FW1 | Sequence walking primer for lambda clones. | CGACTGGGTTCATTAGGGAC |
| o*Eg*λU3U5A-U3FW2a | Sequence walking primer for lambda clones. | GTATTCAGTGCTCATCAAGGCG |
| o*Eg*λU3U5A-U3FW2b | Sequence walking primer for lambda clones. | AACATTCGGTCCATGGTGTTGGTGGC |
| o*Eg*λU3U5A-U3RW1 | Sequence walking primer for lambda clones. | ACCATGGACCGAATGTTGCCGTAACC |
| p94 | Used for 3′ RACE.^16^ | AATAAAGCGGCCGCGGATCCAAT_17_V |
| oAR7 | Used for 3′ RACE.^16^ | CCGGAATTCAATAAAGCGGCCGCGGATCCAA |
